# Clustering and prediction of disease progression trajectories in Huntington's disease: An analysis of Enroll-HD data using a machine learning approach

**DOI:** 10.3389/fneur.2022.1034269

**Published:** 2023-01-30

**Authors:** Jinnie Ko, Hannah Furby, Xiaoye Ma, Jeffrey D. Long, Xiao-Yu Lu, Diana Slowiejko, Rita Gandhy

**Affiliations:** ^1^Genentech Inc., South San Francisco, CA, United States; ^2^Roche Products Ltd., Welwyn Garden City, United Kingdom; ^3^University of Iowa Health Care, Iowa City, IA, United States

**Keywords:** Huntington's disease, clustering, trajectory, prediction, machine learning

## Abstract

**Introduction:**

Huntington's disease (HD) is a rare neurodegenerative disease characterized by cognitive, behavioral and motor symptoms that progressively worsen with time. Cognitive and behavioral signs of HD are generally present in the years prior to a diagnosis; however, manifest HD is typically assessed by genetic confirmation and/or the presence of unequivocal motor symptoms. Nevertheless, there is a large variation in symptom severity and rate of progression among individuals with HD.

**Methods:**

In this retrospective study, longitudinal natural history of disease progression was modeled in individuals with manifest HD from the global, observational Enroll-HD study (NCT01574053). Unsupervised machine learning (k-means; km3d) was used to jointly model clinical and functional disease measures simultaneously over time, based on one-dimensional clustering concordance such that individuals with manifest HD (*N* = 4,961) were grouped into three clusters: rapid (Cluster A; 25.3%), moderate (Cluster B; 45.5%) and slow (Cluster C; 29.2%) progressors. Features that were considered predictive of disease trajectory were then identified using a supervised machine learning method (XGBoost).

**Results:**

The cytosine adenine guanine-age product score (a product of age and polyglutamine repeat length) at enrollment was the top predicting feature for cluster assignment, followed by years since symptom onset, medical history of apathy, body mass index at enrollment and age at enrollment.

**Conclusions:**

These results are useful for understanding factors that affect the global rate of decline in HD. Further work is needed to develop prognostic models of HD progression as these could help clinicians with individualized clinical care planning and disease management.

## Introduction

Huntington's disease (HD) is an autosomal dominant neurodegenerative disease caused by a cytosine adenine guanine (CAG) trinucleotide repeat expansion in the huntingtin gene, resulting in the production of the toxic mutant huntingtin protein (1, 2). It is characterized by a triad of cognitive, behavioral and motor symptoms leading to functional decline and progressive loss of independence.

A CAG repeat length of 36–39 shows incomplete penetrance, whereas a CAG repeat length of >39 shows complete penetrance (1–3), which means that individuals will inevitably experience progressive motor, cognitive and functional decline. The time to onset of motor symptoms is inversely correlated to CAG expansion, but this usually occurs in adult life, with a mean age of motor onset between 30 and 50 years (4). Motor symptoms in the early stages of HD include chorea (5).

The average illness course post-motor onset is approximately 15 years (6, 7), with pneumonia, heart failure or other complications frequently cited as the immediate cause of death (8–10). In the later stages of the disease, impairment of voluntary movements is seen, which manifests as symptoms including incoordination, speech difficulties, swallowing difficulties, bradykinesia and rigidity (5).

Individuals with HD can be categorized as having either premanifest disease (genetic confirmation prior to symptom onset) or manifest disease (a clinical diagnosis based on the presence of unequivocal motor signs) (4). There is variability in individuals with HD on how symptom severity and the rate of symptomatic change occur over the course of the disease (11). Expanded CAG size is highly predictive of rate of clinical decline (12), and other important biological and environmental factors such as body mass index (BMI), age, psychiatric comorbidities, concomitant medication and other genetic modifiers have also been found to be correlated with the rate of clinical deterioration (13–15).

The Enroll-HD registry (NCT01574053) is a large, global, longitudinal patient registry which has been widely used to model the natural history of HD (16–18). Data from Enroll-HD represent the natural history of HD based upon the current standard of care in each of the countries it is established in (including from Europe, North America, Latin America, Australia and New Zealand). Over 20,000 participants have enrolled to date, making Enroll-HD the largest ongoing HD registry worldwide (https://www.enroll-hd.org/).

Previous studies, such as that conducted by Ghazaleh et al., used random forest methods to rank the predictive power of key features on single Unified Huntington's Disease Rating Scale (UHDRS) endpoints over a 2-year period (14). The composite UHDRS (cUHDRS) (19) is made up of four UHDRS scales (Total Motor Score [TMS], Total Functional Capacity [TFC], Symbol Digit Modalities Test [SDMT] and Stroop Word Reading [SWR] (19)), and is thought to capture global clinical decline more sensitively than a single measure; however, the cUHDRS does not include a measure of mood, nor does it consider how performance on motor, cognitive and mood domains may vary between individuals and at different times over the disease course. To expand on this, the current study considers the evolution of multiple endpoints jointly and predicts progression of scores for each domain at any given time. The advantage of this multidimensional approach is that it takes into consideration the holistic nature of HD, by capturing the evolution of not just the multiple motor, cognitive and behavioral domains. Compared with studies that pool all patients together in a single progression model, the present study acknowledges the substantial heterogeneity in the progression trajectories of individuals with HD by using a data-driven approach to identify subgroups or clusters of patients that progress at different rates over time.

In the current study, we clustered patients with manifest HD according to their disease trajectories using data from Enroll-HD, to quantitatively describe HD progression trajectories for symptoms associated across multiple clinical domains. After clustering patients into separate trajectory profiles, we identified which features best predicted trajectory assignment using a cross-sectional set of features captured *via* enrollment data at the patient's first Enroll-HD visit.

## Methods

### Study population

The following eligibility criteria were established for this study:

- manifest HD (defined as carriers with clinical features that are regarded in the opinion of the raters as diagnostic of HD at enrollment visit [variable name hdcat_l = 3] which incorporated diagnostic confidence level = 4)
- at least two annual visits including enrollment visit (variable name visitnum = 1)
- age at symptom onset ≥20 (to limit chances of including those with pediatric-onset HD [variable name sxrater ≥20])
- rater can estimate participant's time of symptom onset with high confidence
- subjects with TMS, SDMT and Apathy scores at baseline.

**Figure 1:**
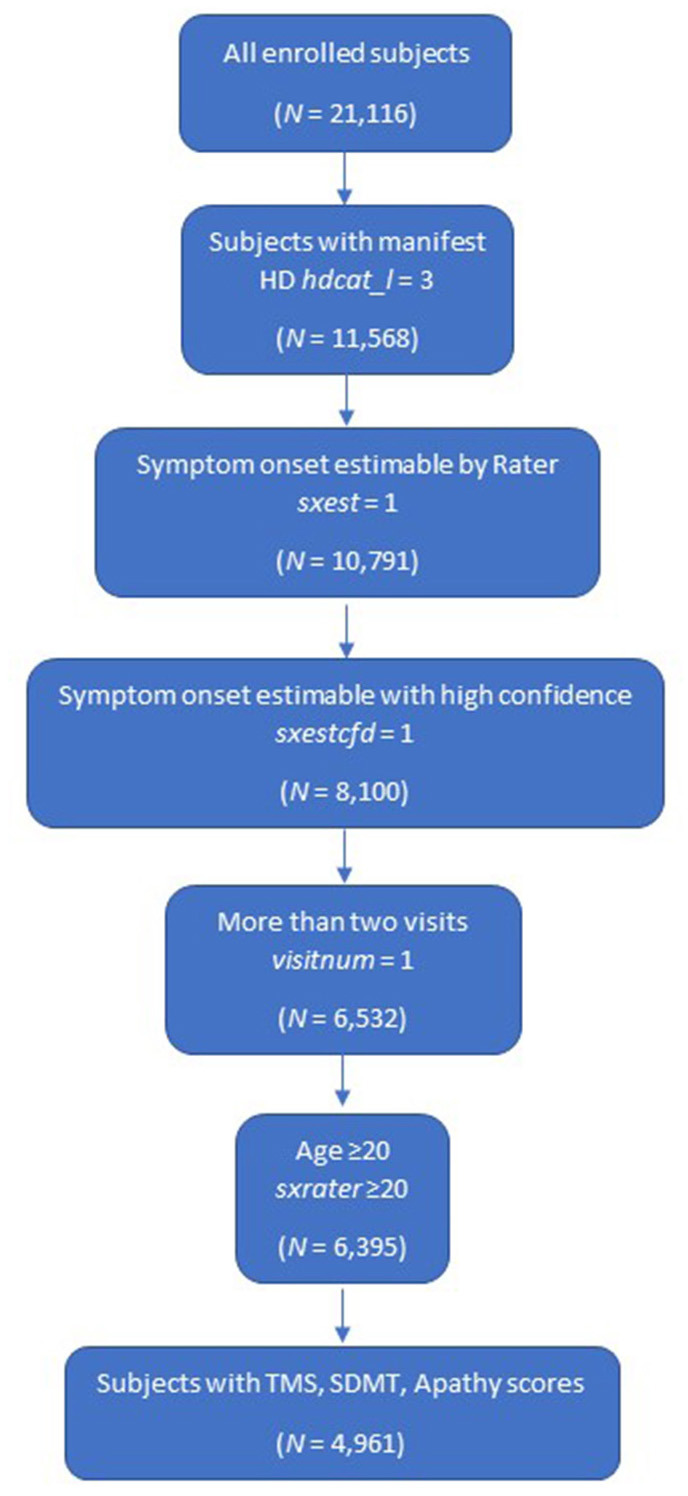
Study population. HD, Huntington's disease; SDMT, Symbol Digit Modalities Test; TMS, Total Motor Score.

Overall, a cohort of 4,961 subjects was included in the k-means clustering model (Figure 1).

### Study design

This was a retrospective study that involved a secondary analysis on data collected from the longitudinal, global observational study (Enroll-HD data PDS5; October 2020). A patient cohort was defined based on their eligibility criteria at their first Enroll-HD study visit (enrollment) and index was defined as the date of first motor symptom onset, as estimated by the study investigator based on all available information. This study focused only on patients aged 0–19 years since symptom onset, based on the number of patients available. Longitudinal trajectories were therefore estimated across a continuous time window, including a period before (symptom onset until enrollment, during which patients were not under observation in the Enroll-HD database) and after enrollment into the study (enrollment until last study visit, where patients were under observation in the Enroll-HD database). The advantage of using symptom onset as the index date is the clinical relevance of this milestone compared with study enrollment, and it allows for a longer follow-up period from which to extrapolate longitudinal progression.

### Data source

Data used in this study were generously provided by the participants in the Enroll-HD study and made available by CHDI Foundation, Inc. Enroll-HD is a global, multicenter, longitudinal, observational study made available *via* a clinical research platform designed to facilitate clinical research in HD (17). Core datasets including HD-specific scales such as the UHDRS assessments, CAG expansion length and demographic and medical history are collected annually from all research participants. Data are monitored for quality and accuracy using a risk-based monitoring approach. All sites are required to obtain and maintain local ethical approval. All assessments were performed by trained clinical personnel. A variety of training methods including practice videos and test assessments were used to train and certify raters. Additionally, manuals were provided to participating sites with instructions for implementing, administering and scoring study instruments. To the extent possible, each site was asked to use the same individual rater to administer study instruments to a particular participant for the duration of the study to maximize internal consistency (https://www.enroll-hd.org/).

### Clustering of disease trajectories

We used a k-means approach to cluster the longitudinal trajectories of participants, based on a statistical index of similarity. The k-means approach is an unsupervised machine learning approach and is used to reduce heterogenous longitudinal data into distinct, homogeneous clusters (20). An optimal number of clusters was identified by non-parametric computations using the Calinski-Harabasz (21) index based on between-cluster and within-cluster variances.

Analysis was carried out first for each individual clinical or social outcome measure, including TFC, TMS, SWR, SDMT, and Problem Behaviors Assessment Short Form (PBA-s) Apathy score. These outcomes were selected due to their clinical relevance in HD with regard to the motor, cognitive and behavioral phenotypes.

Concordance of clustering allocation by individual score was checked against one symptom measurement from each triad of HD symptom domains: cognitive, motor and behavioral domains.

A k-means method for joint trajectories (R package KML 3d) (20) was used for jointly modeling clinical and functional or social outcome measures based on the one-dimensional clustering concordance to modeling motor, cognitive and behavioral trajectories. The 3D clustering was selected as those motor, cognitive and apathy outcomes are related to each other as disease progresses, therefore 3D clustering can replace individual three cluster trajectories. This single 3D cluster was used to build a single prediction model that could predict disease progression trajectories discussed in the next section. TMS (motor domain), SDMT (cognitive domain) and Apathy (behavioral domain) were used to cluster participants based on similarities in progression trajectories. 3D dynamic plots were exported to visualize joint clustering, which provides better representation of the interaction between each pair of two outcome trajectories. The optimal number of clusters was selected based on model selection criteria (to achieve optimal partition) and clinical interpretation.

### Predicting trajectory assignment

The most impactful features that predicted trajectory assignment were identified using supervised learning. The extreme gradient boosting machines algorithm (XGBoost) (21) was used to identify impactful features out of a preselected pool of demographic, genetic/family history, social, symptom, medication and clinical factors based on published literature and clinical input (Table 1). Variable importance scores were calculated from loss of function then scaled from 0 to 1. Scores closer to 0 were indicative of a less important variable in the prediction model; scores closer to 1 were indicative of higher importance.

**Table 1:** Candidate features tested for cluster prediction using a supervised machine learning method (XGBoost).

Predicting feature group*	**Predicting feature descriptions**
Demographic	Region, sex, race, weight at visit, BMI at enrollment visit
Disease characteristics	Age at visit, CAG repeat length, CAP score at enrollment visit, years since rater's diagnosis
Social status at visit	Education level (ISCED), caregiver status
Use of concomitant medication	E.g. use of tiapride, tetrabenazine, olanzapine
Family history	Including mother/father affected status, age of onset of symptoms in mother/father, etc.
HD symptom history	Presence or absence at enrollment visit (depression, irritability, violent behavior, apathy, psychosis, perseverative obsessive behaviors, cognitive impairment)
Suicidal history	Presence or absence at enrollment visit

^*^All information for predicting features was collected at enrollment visit because baseline from Enroll-HD is not a true baseline for manifest HD.

BMI, body mass index; CAG, cytosine adenine guanine; CAP, CAG-age product; HD, Huntington's disease; ISCED, International Standard Classification of Education.

The model training was performed on a training dataset (randomly selected 80% of subjects) and model performance was evaluated on a testing set (the remaining data) with 5-fold cross-validation. Partial dependence plots were produced to describe the marginal effect on target features.

### Handling of missing data

Missing values were interpreted as containing information (i.e., missing for a reason), rather than missing at random, as missed visits were more likely to occur in patients with greater disease severity. During the tree building process, split decisions for every node were found by minimising loss of function and treating missing values as a separate category.

XGBoost automatically learned the most appropriate direction to diverge when a value was missing (21). The raw variable importance for each feature was calculated then scaled between 0 and 1 as relative scales (*via* H2O) were plotted (22).

## Results

### Unsupervised learning: K-mean clustering

Three clusters were selected as the optimal number for compactness within, and separation between, clusters. In addition, the presentation of the clusters in a three-dimensional view (Figures 3–5) provides a view of different modalities (x – cognition, y – time since onset and z – motor) and their relationship to one another.

Concordance was examined to select the scale that best represented the three domains. Concordance rates were high between SWR and SDMT (cognitive), and between TFC (daily function) and TMS (motor). Therefore, SWR and SDMT were considered exchangeable to cluster patients in the cognitive domain. This was similar for TFC and TMS in the motor/daily function domains. Additionally, Apathy score was selected to represent the behavioral domain among PBA-s scores as this had similarities in disease progression trajectories.

**Figure 2:**
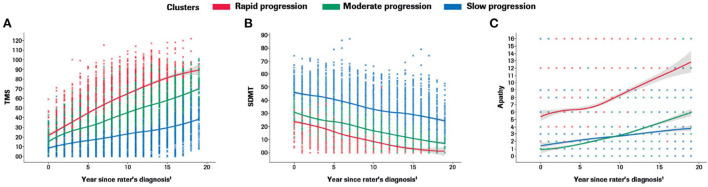
Marginal plots to demonstrate rapid, moderate and slow disease progression trajectories in the motor **(A)**, cognitive **(B)** and behavioral **(C)** domains included in the multidimensional progression model*. *Marginal 2D plots are shown to visualize the longitudinal progression for each endpoint separately; however, a multidimensional approach was used (km3d) where all three endpoints were jointly modeled.^†^Symptom onset time ‘0' defined as the approximate time of first symptom onset (any domain) as determined by the rater using information from multiple sources (e.g., information provided by participant and/or caregiver, medical notes, etc). SDMT, Symbol Digit Modalities Test; TMS, Total Motor Score.

**Figure 3:**
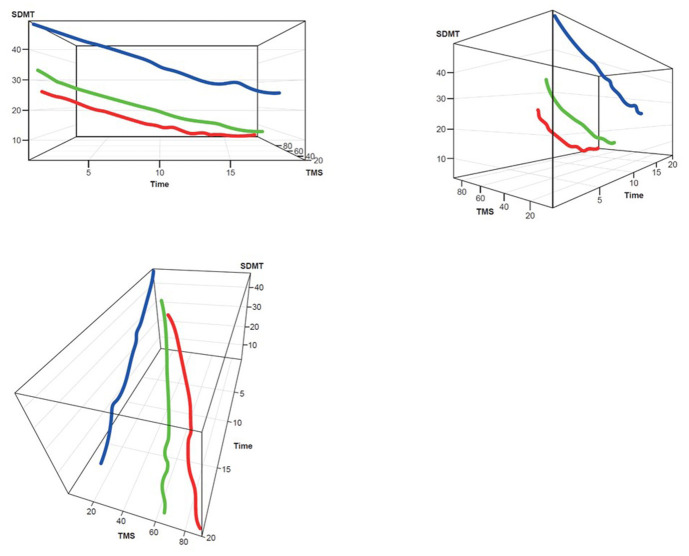
TMS–SDMT 3D plot for clustering. Red, rapid progressors; Green, moderate progressors; Blue, slow progressors; SDMT, Symbol Digit Modalities Test; TMS, Total Motor Score.

**Figure 4:**
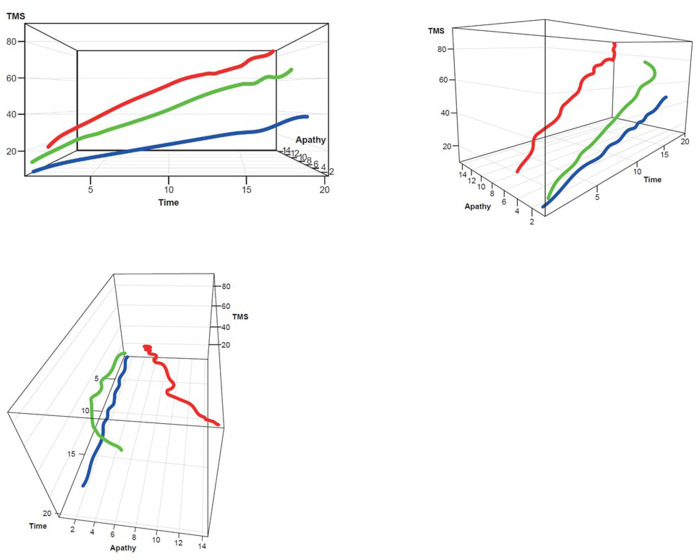
TMS–Apathy 3D plot for clustering. **Red**, rapid progressors; **Green**, moderate progressors; **Blue**, slow progressors; TMS, Total Motor Score.

**Figure 5:**
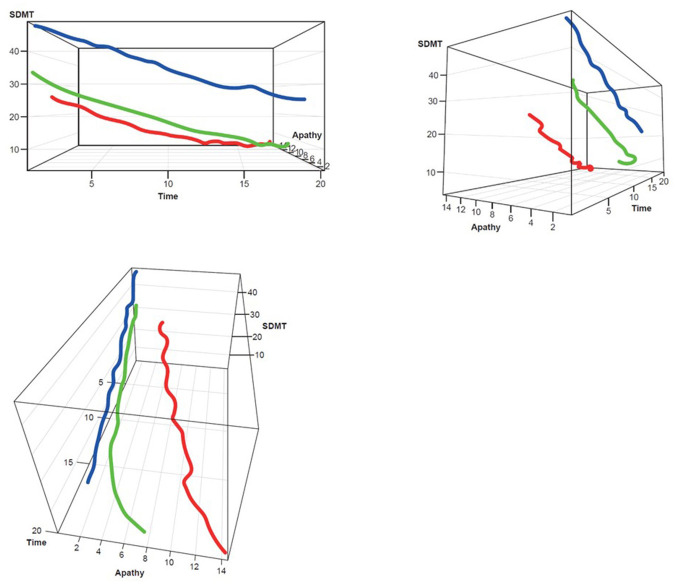
SDMT–Apathy 3D plot for clustering. **Red**, rapid progressors; **Green**, moderate progressors; **Blue**, slow progressors; SDMT, Symbol Digit Modalities Test.

Participants with manifest HD were grouped into three clusters (rapid [Cluster A], moderate [Cluster B] or slow [Cluster C] progressors) based on joint longitudinal trajectories of the selected outcomes: SDMT (cognition), Apathy (behavioral) and TMS (motor) scores (Figures 2–5).

### Demographics of each cluster

Out of 4,961 participants, 1,255 (25.3%) were categorized as rapid progressors, 2,256 (45.5%) were categorized as moderate progressors and 1,450 (29.2%) were categorized as slow progressors. The demographics and clinical characteristics of each cluster and the overall cohort at enrollment visit (first visit) are summarized in Table 2 below. The proportion of males and females was similar across clusters (49% male; 52% female). Strikingly, a higher proportion of those in Cluster A (rapid progressors) came with companions to their visit compared with those in Cluster B (moderate) or C (slow). History of medications including tetrabenazine, antipsychotics and antidepressants, and mood problems such as apathy, irritability and psychosis were more common in Cluster A than Cluster B or C. Higher CAG and CAG-age product (CAP), lower age of parent HD diagnosis and lower level of education were also observed in those in Cluster A. The age of symptom onset and number of years since symptom onset did not seem to vary systematically between clusters.

**Table 2:** Baseline characteristics by cluster.

	**A (*N* = 1,255)**	**B (*N* = 2,256)**	**C (*N* = 1,450)**	**Overall (*N* = 4,961)**
**Sex**				
F	656 (52.3%)	1,178 (52.2%)	706 (48.7%)	2,540 (51.2%)
M	599 (47.7%)	1,078 (47.8%)	744 (51.3%)	2,421 (48.8%)
**Race (pooled)**				
Others	64 (5.1%)	130 (5.8%)	64 (4.4%)	258 (5.2%)
White	1,191 (94.9%)	2,126 (94.2%)	1,386 (95.6%)	4,703 (94.8%)
**Region (pooled)**				
Europe	1,066 (84.9%)	1,472 (65.2%)	937 (64.6%)	3,475 (70.0%)
Northern America	153 (12.2%)	684 (30.3%)	461 (31.8%)	1,298 (26.2%)
Others	36 (2.9%)	100 (4.4%)	52 (3.6%)	188 (3.8%)
**Came with companions or not**				
No	179 (14.3%)	672 (29.8%)	695 (47.9%)	1,546 (31.2%)
Yes	1,072 (85.4%)	1,578 (69.9%)	755 (52.1%)	3,405 (68.6%)
Missing	4 (0.3%)	6 (0.3%)	0 (0%)	10 (0.2%)
**Nerve drug**				
No	775 (61.8%)	1,628 (72.2%)	1,225 (84.5%)	3,628 (73.1%)
Yes	480 (38.2%)	628 (27.8%)	225 (15.5%)	1,333 (26.9%)
**Tetrabenazine flag: trt movement disorder by HD**				
No	802 (63.9%)	1,728 (76.6%)	1,278 (88.1%)	3,808 (76.8%)
Yes	453 (36.1%)	528 (23.4%)	172 (11.9%)	1,153 (23.2%)
**Antipsychotics-Benzamides flag: depression drug**				
No	916 (73.0%)	1,975 (87.5%)	1,321 (91.1%)	4,212 (84.9%)
Yes	339 (27.0%)	281 (12.5%)	129 (8.9%)	749 (15.1%)
**Antipsychotics flag: bipolar disorder drug**				
No	624 (49.7%)	1,629 (72.2%)	1,163 (80.2%)	3,416 (68.9%)
Yes	631 (50.3%)	627 (27.8%)	287 (19.8%)	1,545 (31.1%)
**Mother affected**				
No	786 (62.6%)	1,354 (60.0%)	773 (53.3%)	2,913 (58.7%)
Yes	469 (37.4%)	902 (40.0%)	677 (46.7%)	2,048 (41.3%)
**Father affected**				
No	821 (65.4%)	1,436 (63.7%)	884 (61.0%)	3,141 (63.3%)
Yes	434 (34.6%)	820 (36.3%)	566 (39.0%)	1,820 (36.7%)
**Suicide attempt**				
No	809 (64.5%)	1,657 (73.4%)	970 (66.9%)	3,436 (69.3%)
Yes	445 (35.5%)	598 (26.5%)	479 (33.0%)	1,522 (30.7%)
Missing	1 (0.1%)	1 (0.0%)	1 (0.1%)	3 (0.1%)
**Depression history**				
No	210 (16.7%)	662 (29.3%)	339 (23.4%)	1,211 (24.4%)
Yes	1,045 (83.3%)	1,593 (70.6%)	1,111 (76.6%)	3,749 (75.6%)
Missing	0 (0%)	1 (0.0%)	0 (0%)	1 (0.0%)
**Irritability history**				
No	238 (19.0%)	732 (32.4%)	380 (26.2%)	1,350 (27.2%)
Yes	1,016 (81.0%)	1,523 (67.5%)	1,070 (73.8%)	3,609 (72.7%)
Missing	1 (0.1%)	1 (0.0%)	0 (0%)	2 (0.0%)
**Violent or aggressive history**				
No	619 (49.3%)	1,441 (63.9%)	845 (58.3%)	2,905 (58.6%)
Yes	635 (50.6%)	815 (36.1%)	605 (41.7%)	2,055 (41.4%)
Missing	1 (0.1%)	0 (0%)	0 (0%)	1 (0.0%)
**Apathy history**				
No	80 (6.4%)	929 (41.2%)	587 (40.5%)	1,596 (32.2%)
Yes	1,175 (93.6%)	1,327 (58.8%)	863 (59.5%)	3,365 (67.8%)
**Perseverative obsessive behaviors history**				
No	315 (25.1%)	963 (42.7%)	707 (48.8%)	1,985 (40.0%)
Yes	939 (74.8%)	1,293 (57.3%)	743 (51.2%)	2,975 (60.0%)
Missing	1 (0.1%)	0 (0%)	0 (0%)	1 (0.0%)
**Psychosis (hallucinations or delusions) history**				
No	1,008 (80.3%)	2,028 (89.9%)	1,329 (91.7%)	4,365 (88.0%)
Yes	247 (19.7%)	228 (10.1%)	121 (8.3%)	596 (12.0%)
**Family history of psychotic illness**				
No	169 (13.5%)	169 (7.5%)	82 (5.7%)	420 (8.5%)
Yes	40 (3.2%)	26 (1.2%)	27 (1.9%)	93 (1.9%)
Missing	1,046 (83.3%)	2,061 (91.4%)	1,341 (92.5%)	4,448 (89.7%)
**Cognitive impairment or dementia history**				
No	302 (24.1%)	1,012 (44.9%)	781 (53.9%)	2,095 (42.2%)
Yes	952 (75.9%)	1,242 (55.1%)	669 (46.1%)	2,863 (57.7%)
Missing	1 (0.1%)	2 (0.1%)	0 (0%)	3 (0.1%)
**BMI**				
Mean (SD)	24.8 (4.74)	24.8 (4.61)	26.2 (5.18)	25.2 (4.86)
Median [Min, Max]	24.2 [14.9, 52.6]	24.1 [11.7, 49.5]	25.3 [16.0, 58.3]	24.5 [11.7, 58.3]
Missing	48 (3.8%)	44 (2.0%)	20 (1.4%)	112 (2.3%)
**CAG**				
Mean (SD)	44.6 (3.74)	43.9 (3.31)	42.8 (2.61)	43.7 (3.31)
Median [Min, Max]	44.0 [36.0, 64.0]	43.0 [36.0, 63.0]	42.0 [36.0, 58.0]	43.0 [36.0, 64.0]
**CAP: age diagnosis** *(CAG high −**33.6)**				
Mean (SD)	526 (91.6)	512 (84.1)	439 (84.0)	494 (93.1)
Median [Min, Max]	522 [161, 1,050]	509 [152, 1,070]	442 [91.3, 748]	496 [91.3, 1,070]
**Mother symptom onset age**				
Mean (SD)	45.5 (12.5)	47.0 (12.2)	49.0 (11.5)	47.3 (12.1)
Median [Min, Max]	45.0 [20.0, 85.0]	45.0 [19.0, 90.0]	50.0 [21.0, 90.0]	46.0 [19.0, 90.0]
Missing	786 (62.6%)	1,354 (60.0%)	773 (53.3%)	2,913 (58.7%)
**Father symptom onset age**				
Mean (SD)	47.3 (11.4)	48.5 (12.0)	49.9 (11.6)	48.7 (11.8)
Median [Min, Max]	45.5 [17.0, 80.0]	48.0 [10.0, 87.0]	50.0 [18.0, 85.0]	49.0 [10.0, 87.0]
Missing	821 (65.4%)	1,436 (63.7%)	884 (61.0%)	3,141 (63.3%)
**Years since symptom onset**				
Mean (SD)	5.51 (3.76)	5.96 (4.28)	6.30 (5.22)	5.95 (4.47)
Median [Min, Max]	5.00 [−3.00, 17.0]	5.00 [−4.00, 20.0]	6.00 [−5.00, 21.0]	5.00 [−5.00, 21.0]
**Education level (pooled)**				
Advanced (4–6)	378 (30.1%)	1,029 (45.6%)	857 (59.1%)	2,264 (45.6%)
Primary (0–3)	872 (69.5%)	1,218 (54.0%)	592 (40.8%)	2,682 (54.1%)
Missing	5 (0.4%)	9 (0.4%)	1 (0.1%)	15 (0.3%)
**Age of symptom onset**				
Mean (SD)	46.1 (11.9)	47.1 (11.8)	44.1 (10.6)	46.0 (11.5)
Median [Min, Max]	46.0 [20.0, 81.0]	47.0 [20.0, 85.0]	45.0 [20.0, 75.0]	46.0 [20.0, 85.0]

BMI, body mass index; CAG, cytosine adenine guanine; CAP, CAG-age product; F, Female; HD, Huntington's disease; M, male; SD, standard deviation.

Cluster A, rapid progressors, Cluster B, moderate progressors, Cluster C, slow progressors.

The majority of participants (>94%) were white regardless of clustering and most participants were from Europe for all clusters. Compared with Clusters B and C, Cluster A (rapid progressors) included a higher proportion of Europeans and a lower proportion of Northern Americans.

### Predicting features

**Figure 6:**
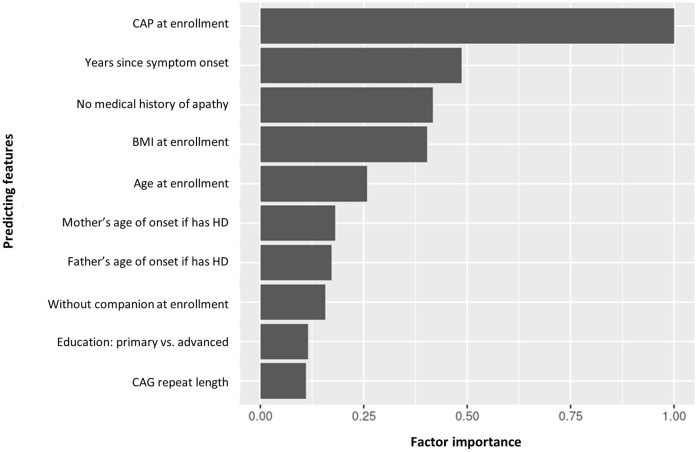
Factor importance of the top 10 predicting features of manifest HD progression clusters. BMI, body mass index; CAG, cytosine adenine guanine; CAP, CAG-age product; HD, Huntington's disease.

The top predicting features of cluster assignment can be seen in Figure 6. CAP at enrollment visit was the top predicting feature to predict cluster assignment, followed by years since symptom onset, no medical history of apathy, BMI and age at enrollment visit.

**Figure 7:**
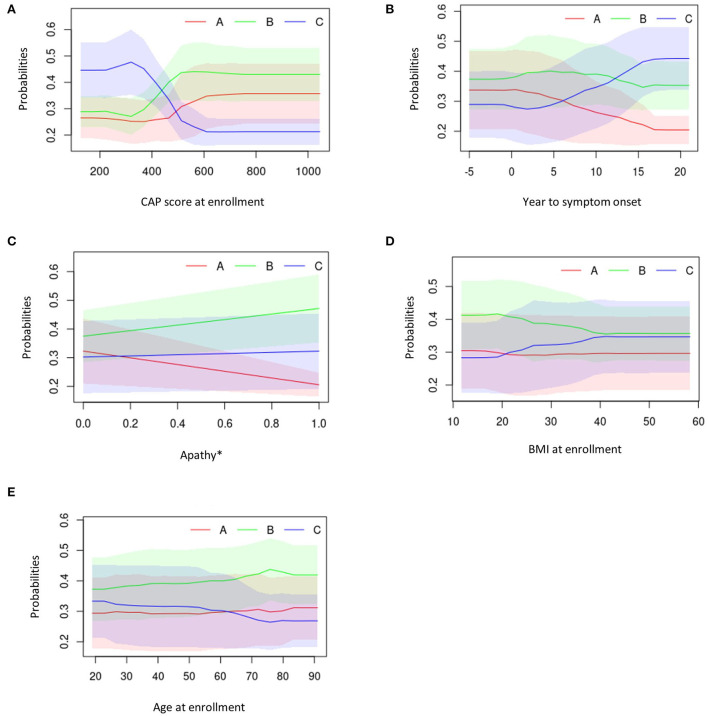
Partial dependency plots describing the top five predicting features against average cluster probability (y-axis). **(A)** CAP score at enrollment. **(B)** Years to symptom onset. **(C)** Apathy. **(D)** BMI at enrollment. **(E)** Age at enrollment. *0 = No; 1 = Yes. BMI, body mass index; CAG, cytosine adenine guanine; CAP, CAG-age product.

For the 10 top predicting features, the partial dependence plots provided a graphical explanation of the marginal effect of each feature on clusters. The top five predicting features are illustrated in Figures 7A–E. The other partial dependency plots can be seen in Supplementary Figures 1A–E.

Overall, participants in Cluster C exhibited lower CAP scores at enrollment visit, longer duration between symptom onset and enrollment visit, presence of medical history of apathy, higher BMI scores and younger age at enrollment visit (Figures 7A–E).

Those in Cluster B had the highest CAP score at enrollment visit, a moderate duration between symptom onset and enrollment visit, higher medical history of apathy, higher BMI scores and were older at enrollment (Figures 7A–E).

Participants in Cluster A exhibited moderate CAP scores at enrollment visit, had a short duration between symptom onset and enrollment visit, low medical history of apathy, had the lowest BMI at enrollment and were older at enrollment visit (Figures 7A–E).

## Discussion

The natural history of HD varies greatly among individuals, particularly since motor, cognitive and behavioral symptoms may manifest differently across individual patients over time. The present study applies k-means clustering to the Enroll-HD data, which to our knowledge has not been applied to explore the natural progression of HD before. This machine learning method, coupled with a multivariate approach, enables us to consider change over time simultaneously among motor, cognitive and behavioral domains. In doing so, we found that it was possible to crudely cluster patients into rapid, moderate and slow progressors, and to identify a set of cross-sectional characteristics that best predicted which trajectory they would follow. These results provide useful additions to the HD natural history literature.

Participants with manifest HD were clustered based on joint longitudinal trajectories of TMS, SDMT and Apathy scores. Apathy was selected as this score is most commonly associated with HD among behavioral domains and was also available in the Enroll-HD data. Additionally, the Apathy scores collected show trends over time, which was an advantage over other behavioral domains. At the time of enrollment, CAP score was the most impactful feature predictive of manifest HD progression cluster assignment, followed by years since symptom onset and medical history of apathy. Clustering and prediction provide a profile for different rates of manifest HD progression, which may be useful to guide personalized clinical care and management plans.

Holistically capturing disease progression can be helpful for understanding the global pattern of symptoms over the lifespan of individuals with HD. There have been other efforts to simultaneously capture the progression of multiple outcomes. For example, the cUHDRS (23) is a composite clinically validated measure that weights the SDMT, SWR, TMS and TFC into a single component score. Whilst the cUHDRS showed good utility as a primary outcome measure in global clinical trials, in the present study we wanted to acknowledge the behavioral/psychiatric domain of HD and to be able to visualize how each domain evolves over time. A study utilizing a principal component analysis on longitudinal data from the TRACK-HD and Track-ON studies found that the first principal component correlated highly with all motor–cognitive measures, accounting for 67.6% of their combined variance and was inversely and non-linearly associated with age of onset and CAG repeat length (included in the top 10 features in our study) (12). Whilst a principal component analysis approach is a good way to reduce multidimensional data, it is also difficult to interpret the influence of the individual component features. In our study we selected a priori: a set of clinically meaningful motor, cognitive and behavioral outcomes that clinicians were likely to be familiar with in clinical practice, and we showed the joint evolution of these outcomes in multiple dimensions, which aids interpretability. Despite the individual heterogeneity in the rate of decline between domains, our approach suggests that it may be possible to classify patients into rapid, moderate and slow progressors, depending on their overall function.

By assessing the baseline demographics and predictors of cluster assignment, it is evident that those in Cluster A are more advanced at enrollment than those in Cluster B or C, since they have a longer history of medication use and behavioral issues. Interestingly, we observe that patients in this cluster have a lower educational attainment and their parents are diagnosed at a younger age. CAG repeat length is also longer in patients in Cluster A. All together, this supports the understanding that familial factors may influence the rate of progression. These familial factors may include a combination of genetic factors (e.g., inherited CAG repeat length which is inversely associated with rate of progression (24)) and environmental factors (e.g., generational burden which may influence the ability to receive education). It was not in the scope of this analysis to comment on the causal relationship between genetic and environmental factors and symptomatic therapies, and their effect on longitudinal outcomes; however, our findings corroborate a picture of higher disease burden in those who progress more rapidly. Indeed, despite methodological differences, our study supports other publications that show that the CAP score (also known as the disease burden score) and CAG repeat length are within the top 10 predicting factors of longitudinal HD progression (14).

Machine learning approaches such as this could be considered for application in real-world clinical practice to support the treating physician in assessing whether patients are improving or worsening on disease-modifying treatment (DMT) compared with patients of a similar clinical profile receiving standard of care. For example, a clinical visit could include a series of core assessments (equivalent to the enrollment visit in this study), using a model that is trained on Enroll-HD data, which could predict which trajectory a patient is on compared with others with a similar profile. Over time, this prediction may support a change in clinical decision making, thereby personalising each patient's healthcare journey. In addition to the TMS, SDMT and PBA-s Apathy outcomes, the top 10 predictive features in this study may be considered as high-priority variables to measure at a clinical visit, in order to maximize the chances of successfully predicting performance. DMTs are in development that aim to slow or halt the progression of HD, although a clinician-facing tool for assessing real-world clinical efficacy is currently not available. A clinical dashboard has been developed to compare an individual's progression with a global cohort adjusting for age and CAG repeat, utilizing Enroll-HD data (18). However, the heterogeneity in the progression of patients was not accounted for, and potential predictors other than age and CAG repeat were not examined. Nevertheless, their findings, like ours, support the notion that providing clinicians with the ability to monitor an individuals' progression against key cognitive, behavioral and motor symptoms in real time will support enhanced decision making and identify those eligible for clinical trials of DMTs earlier.

The premanifest participants were not included as this study focuses on the disease trajectory from first motor symptom onset. However, it will be of interest to build additional predictive models in future studies to estimate first symptom onset time using the premanifest participants' baseline or disease characteristics.

Our results should be interpreted with caution due to the following limitations. We chose ‘estimated symptom onset' as the study index date, since it is a more clinically relevant disease milestone than the first Enroll-HD visit. It should be acknowledged however, that there is likely some uncertainty around the exact symptom onset as this is a retrospective estimate by the person conducting the Enroll-HD assessment, since symptom onset was not observed during the course of the Enroll-HD data. This enabled us to describe trajectories over a longer time period (~20 years) even though they were not continually observed during this time. To address this concern, the information for years from symptom onset to enrollment visit was included in the prediction model. Other modifiers of progression have been identified previously, including other genetic modifiers (25), regional brain atrophy (12) and lifestyle factors (26). However, these data were not available in the Enroll-HD periodic dataset and therefore it was not possible to include the list of features explored in this study.

In conclusion, knowledge of predictive features of manifest HD progression could guide the development of individualized clinical care and disease management approaches. Future research could extend this work by applying data-driven machine learning models to capture disease progression across multiple domains. Such a tool could be used in a real-world setting to understand whether patients are responding positively to treatments relative to natural history.

## Data availability statement

The data analyzed in this study were obtained from Enroll-HD, the following licenses/restrictions apply: Researchers at recognized research organizations can request data after signing a Data Use Agreement. Requests to access these datasets should be directed to Enroll-HD, https://enroll-hd.org/for-researchers/access-data-biosamples/.

## Ethics statement

Ethical review and approval were not required for the study on human participants in accordance with the local legislation and institutional requirements. Written informed consent for participation was not required for this study in accordance with the national legislation and the institutional requirements.

## Author contributions

JK: planning the data analysis, analyzing the data, and manuscript editing. HF: study design, interpretation, and manuscript editing. XM, X-YL, and RG: research analysis methods, interpretation, and manuscript editing. JDL and DS: planning and interpretation of the analysis and manuscript editing. All authors contributed to the article and approved the submitted version.
