# Supplementary material for: Clustering and prediction of disease progression trajectories in Huntington's disease: An analysis of Enroll-HD data using a machine learning approach
Source: Front Neurol. 2023 Jan 30;13:1034269. doi: 10.3389/fneur.2022.1034269 (PMC9923354; doi:10.3389/fneur.2022.1034269)
Supplement: Supplementary file 1 [file Data_Sheet_1.docx]

Clustering and prediction of disease progression trajectories in Huntington’s disease: An analysis of Enroll-HD data using a machine learning approach

**Figure S1.** **Partial dependency plots describing the bottom five predicting features.** A. Mother’s age if has HD; B. Father’s age if has HD; C. Participants without companions at enrollment; D. Education status; E. CAG repeat length.

**B**

**A**


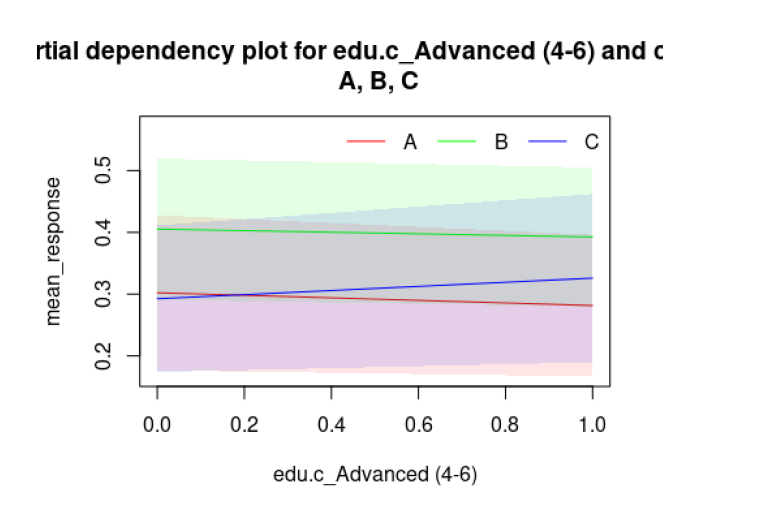

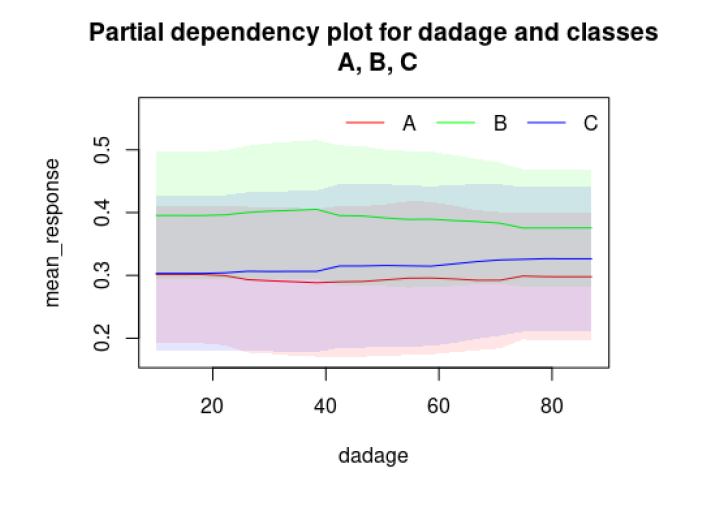

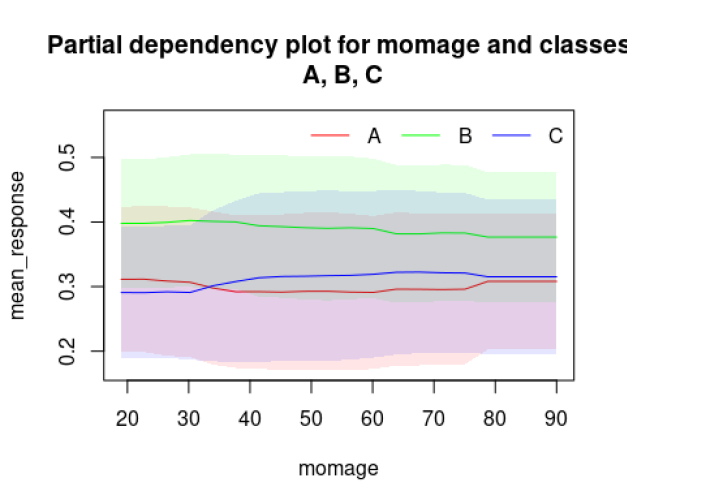


Education: primary vs advanced*

Without companion at enrollment*

Mean response

Mean response

**D**

**C**

Mean response

Father’s age

Mean response

Mother’s age


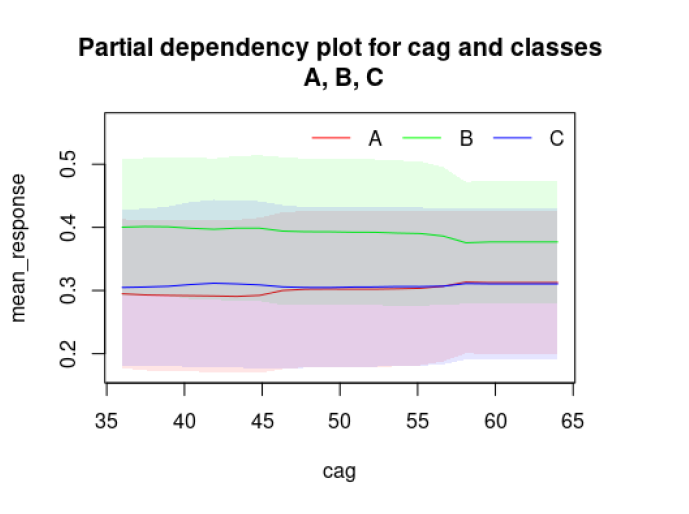

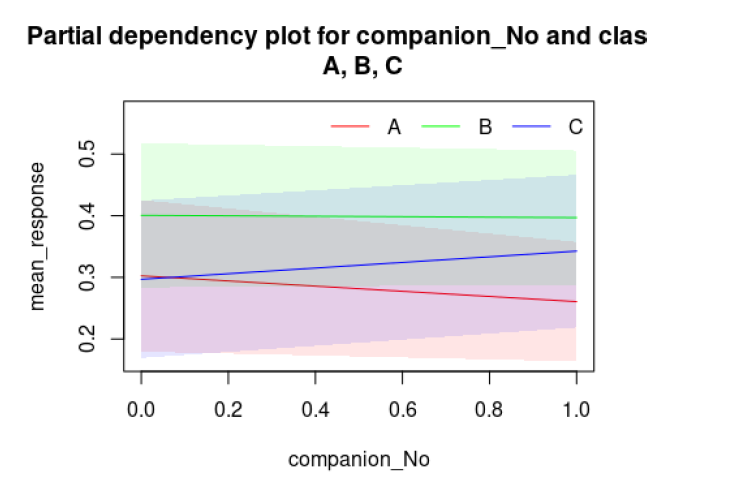


**E**

CAG repeat length

Mean response
